# Incidence and determinants of severe maternal outcome in Jimma University teaching hospital, south-West Ethiopia: a prospective cross-sectional study

**DOI:** 10.1186/s12884-018-1879-x

**Published:** 2018-06-20

**Authors:** Wondimagegnehu Sisay Woldeyes, Dejene Asefa, Geremew Muleta

**Affiliations:** 1 Deparment of Obstetrics and Gynecology, Tercha General Hospital, Tercha, Ethiopia; 2 Deparment of Obstetrics and Gynecology, College of Medicine and Health Sciences, Hawassa University, Hawassa, Ethiopia; 3 Department of Obstetrics and Gynecology, College of Health Sciences, Jimma University, P. O. Box:378, Jimma, Ethiopia; 4 Department of Statistics, College of Natural Science, Jimma University, P. O. Box:378, Jimma, Ethiopia

**Keywords:** Severe maternal morbidity, Severe maternal outcome, WHO, Maternal near-miss, Maternal death, Jimma

## Abstract

**Background:**

Investigating cases of severe maternal morbidity (SMM) and severe maternal outcome (SMO) and the quality of maternal health care using near-miss approach has become popular over recent years. The aim of this study was to determine facility based incidence and the determinants of severe maternal outcome (SMO) using this approach.

**Methods:**

Prospective cross-sectional study among all mothers who presented to study facility while pregnant, during child birth and/or within 42 days after termination of pregnancy seeking care and found to have SMM and SMO during the study period was carried out.

**Results:**

There were total of 2737 live births, 202 SMM and 162 SMO (138 maternal near-misses (MNM) and 24 maternal deaths (MD)) cases. The SMO ratio was 59.2 per 1000 live births and the MNM mortality ratio, mortality index (MI) and maternal mortality ratio (MMR) were: 5.8:1, 14.8% and 876.9 per 100,000 live births respectively. Close to three-fourth of all women with SMO had evidence of organ dysfunction on arrival or within 12 h of hospitalization. The commonest underlying causes for SMO were uterine rupture 27%, followed by hypertensive disorders 24% and obstetric hemorrhage 24%. The highest case fatality rate was found to be associated with eclampsia 28%. Maternal age, residential area, educational status and occupation were associated with SMO (*P* < 0.0001). On binary multivariable logistic regression the occurrence of any delay, intrapartal detection of complication, the mode of delivery and duration of hospitalization had statically significant association with SMO (*p* < 0.05). Optimal number of antenatal care (ANC) visits and delivery by emergency cesarean section (C/S) were found to be protective of SMO.

**Conclusion:**

The occurance SMO in the facility thus in the population served was high. Most of these factors associated with SMO are modifiable; some amenable to social change and the others are within the control of the health system. Thus the finding of this research calls for planning for such changes which can enhance timely and proper detection and management of pregnancy related complications.

## Background

Improving maternal health has received recognition at the global level as evidenced by its inclusion in the Millennium Development Goals (MDGs) with goal to reduce maternal mortality by three fourth by the end of 2015 [1, 2]. The maternal health issue has also continued to be one of top post-2015 sustainable development goal (SDG) agendas and a target for maternal mortality reduction was set [3]. Though, reduction in maternal mortality has traditionally been used as a critical measure of maternal health, it represents only a “tip of the iceberg” of the burden the maternal morbidity and resulting short and long-term sufferings. For every woman who dies of pregnancy-related causes, 20 or 30 others experience acute or chronic morbidity [2]. Therefore relying solely on maternal mortality to assess maternal health overlooks pregnancy continuum from normal to death. In that continuum, pregnancy may be uncomplicated or complicated. Complications range in severity from minor morbidity to potentially life-threatening conditions (PLTC) and life-threatening complications (LTC) [4–7].

In life-threatening pregnancy related complication the woman has one of the two SMO: she may die (maternal deaths) or narrowly escape death (maternal near-miss) cases [5]. Maternal death is defined as death of women during the time of pregnancy, labor and delivery or within the first 42 days after delivery/termination of pregnancy, irrespective of the duration and the site of the pregnancy, from any cause related to or aggravated by the pregnancy or its management, but not from accidental or incidental causes. On the other hand, maternal near-miss case is defined as “a woman who nearly died, but survived a complication that occurred during pregnancy, childbirth, or within 42 days of termination of pregnancy”. Practically, women are considered near miss when they survive organ dysfunction [5, 6].

Women who survive life-threatening conditions (MNM cases) have many aspects in common with those who die of such complications. The assumption is that, a woman suffering from a near miss event is exactly like the one who died, except for the outcome [7–9]. Similar to the case of MD most cases of SMM are preventable. This SMM cases can also be considered a near miss for maternal mortality because without identification and treatment, in some cases, these conditions would lead to maternal death. Thus, the identifications of SMM is crucial for preventing complications that led to mortality and for highlighting ways and opportunities to avoid similar cases in the future.

This has stimulated an interest in investigating cases of SMM in the last few decades and led to the development of the near-miss approach for maternal health [5, 6]. This approach has been found to be useful for the identification of health systems failures and a relevant source of information for policy makers in the selection of maternal health care priorities. Among other positive characteristics, investigating near miss events allows calculation of comparative indices and quantification of intensive care requirements. It also gives the opportunity to interview the woman, as she survived, providing valuable information on the risk factors and substandard care she received. Moreover, investigating the care received may be less threatening to providers because the woman survived [5, 9, 11].

In recent years the near-miss approach for maternal health is being widely used and found to be a valuable tool in, among others, understanding local patterns of maternal mortality and morbidity, strengths and weaknesses in the referral system, and the use of clinical and other health-care interventions [5, 6, 11]. Despite its wide application, there have been challenges with its use mainly due to absence of universal criteria for identification of cases. Criteria for MNM identification were developed by experts from WHO working group for maternal health so as to standardize detection of MNM cases. This technique of MNM identifications proposed by WHO is a two-step process. First; maternal cases with potentially life-threatening conditions, which may or may not be near-miss cases (e.g. specific complications such as severe preeclampsia and/or critical interventions such as blood transfusion) are identified and then identification of near-miss cases based on organ system dysfunction and organ-dysfunction proxies including clinical, laboratory and management criteria [5, 12]. This criterion is validated in different setups and its applicability depends on the local context and availability of resources [12, 13].

In Ethiopia also, MNM review is considered one of the many strategies to tackle the high maternal mortality [6, 14]. The national Maternal Death Surveillance and Response (MDSR) system recommends all MDs and 50% of MNM cases be reviewed in hospital facilities [6]. However, there is limited experience with the use of near-miss approach in Ethiopia and there are few studies available. Specifically, MMR in the study facility, Jimma University Teaching Hospital (JUTH), is very high, 888.5 per 100,000 live births [15] in a review 10 years back. The reason for this high MMR is not studied and there is no study on MNM in JUTH.

This study therefore has determined the incidence and determinants of SMM and SMO in the study facility. In addition, the quality of maternal health care provided at JUTH was assessed using WHO near-miss approach and calculated indicators recommended by WHO. It is the first prospective cross-sectional study conducted to assess cases of MNM and MD in the obstetric practice of southwest part of Ethiopia.

## Methods

### Study settings and design

A prospective cross sectional study was conducted in JUTH from the 1st of February to the 30th of August 2015. JUTH is located in Jimma town, 356 km south-west of Addis Ababa. It is the only referral hospital in south-west Ethiopia with catchment population of over 15 million. Therefore the hospital serves complicated cases referred from 114 health centers and over 18 hospitals (none with intensive care unit (ICU) service) in its catchment. JUTH is also one of the oldest teaching hospitals in the country for undergraduate and post-graduate clinical specialty training in various disciplines including Obstetrics and Gynecology.

The Department of Obstetrics and Gynecology of JUTH has two inpatient wards: Gynecology ward and Obstetrics (maternity, labor and delivery ward which also includes maternity operation theatre) with a total of 80 beds. There is one major operation theatre for Gynecology cases with two operating tables and shared with the general surgery unit. There is an ICU with six beds and three functional mechanical ventilators in the hospital at the time of this study. The ICU is shared by medical, neurological, surgical, pediatrics, orthopedics and obstetric patients. Thus, there is no separate ICU for obstetrics patients.

### Case selection and sample size

All women presenting to the hospital seeking health care during pregnancy, labor and delivery and/or within 42 days after delivery/termination of pregnancy were used as source population. These women were screened for the presence of any severe maternal morbidity and severe maternal outcome (maternal near-miss and deaths). The identification of SMM was based on diagnostic categories such as obstetrical hemorrhage, hypertensive disorders, sepsis or severe systemic infection, uterine rupture, early pregnancy complications and/or other indirect causes. The terms potentially life-threatening conditions, severe maternal complications and severe maternal morbidity were used interchangeably throughout this article.

MNM cases were identified using WHO clinical, laboratory and/or management criteria for organ dysfunction. Mothers with renal dysfunction/failure and requiring dialysis were referred to Addis Ababa and these women were included as near-miss cases in this study. In addition, all mothers who died in the hospital while pregnant, during labor and delivery or within 42 days after delivery/termination of pregnancy and those who were not alive on arrival to the hospital were included in this study.

The sample size was determined using single population proportion formula and it was calculated considering the severe maternal morbidity rate of 310/1000 [16] with 5% significance (i.e. *P* = 0.31, ᾱ = 0.05, margin of error (d) =0.05), and 10% none response rate. Finally, a total of 364 cases with PLTCs or life-threatening complications (LTCs) were analyzed.

### The variables studied were

#### Background information

Age, level of education, occupation, marital status, place of residence and estimated distance of their place of residence from JUTH.

#### Obstetrics variables

Gravidity, whether the woman had ANC visits, number of visits and the place of ANC during the index pregnancy. We considered ANC is optimal if a woman has at least four prenatal visits or in case of women presenting at earlier gestation with complications, having minimum number of ANC appropriate for the duration of pregnancy. In addition, state of pregnancy at the time of first detection of complication is documented to all cases.

#### Facility episode

Date and time of admission and discharge, main reason/symptom for admission, condition/status of the patient and whether she came referred from another institution, mode of transportation used and initiation of treatment on referral and its appropriateness were assessed. Duration of labor and final mode of delivery were recorded for those who arrived before giving birth. In addition, primary underlying causes for PLTC and their temporal pattern of development were documented for all cases. Facility management, including mode of delivery, use of critical interventions, laboratory abnormalities and duration of hospital stay are documented.

The purpose of every maternal near-miss and/or death investigation is to determine its preventability. Preventable/modifiable contributors for SMO were specified according to the three delay model: patient/family factors (health seeking (delay one), transport access (delay two), health system related (delay three) [6]. Thus, the approach recommended by Ethiopian Ministry of Health MDSR guideline to assess preventability of SMO by identifying the contributory factors was used. We identified these factors using the operational definition and presented the detail in this article.

The maternal outcome is documented during the hospital stay or upon discharge. Women with organ dysfunction were categorized as cases of SMO which was sub-categorized as either MNM if they survive the condition or MD if they die and other women without organ dysfunction were classified as SMM cases.

### Data collection tool and procedures

The data collection tool (a structured questionnaire) was developed from the WHO near-miss and the Ethiopian MDSR tools [5, 6]. The MNM approach assesses the clinical care and related interventions; whereas, the MDSR approach explores contributory factors (delay factors) in addition to the clinical care. Patient charts, delivery and operation registration books were used as source of information to complete the questionnaire. In the case of detecting incomplete information from the patients’ and/or facilities’ document; face-to-face interview with the women and/or their family (e.g. if she is dead) was employed to collect the required information after securing an informed consent.

### Data management and analysis

The data was entered in to computer database using Statistical Package for Social Sciences for windows version 20 on a daily basis and finally analyzed using both descriptive and inferential statistics. Regression analysis was used to examine the association between dependent and independent variables. To assess the effects of each independent variable on the outcome variables multivariate logistic analysis was carried out and a 푃 value < 0.25 were taken as indicator for multivariate model. Finally, the findings of the study are presented using tables and graphs.

### Ethical considerations

Ethical clearance was secured from ethical review board of Jimma University College of Health Sciences. Written consent was obtained from every study subject and/or caregivers, in case interview needed by explaining the objective of this research. Any personal identifiers were omitted and the interview was conducted in private places.

## Result

### Socio-demographic and clinical characteristics

There were a total of 2737 live births in the study facility during the study period. A total of 364 women with SMM and SMO (MNM and MD) were identified and analyzed. Two hundred and two of them had SMM making its incidence ratio of 73.8/1000 live births; whereas, the rest 162 cases had LTCs which resulted in SMO (138 of the cases of MNM and 24 MD). The SMO and MNM incidence ratio were 59.2 and 50.4 per 1000 live births respectively and the MMR was 876.9 per 100,000 live births. The MNM to MD ratio and the MI (ratio of MD among women with LTCs) were 5.8:1 and 14.8% respectively.

The socio-demographics and obstetric characteristics of the cases are presented in Table 1. The average age of all women (SMM and SMO) was 27.3 ± 5.5 years and most 205(56%) of them were aged between 25 to 34 years. The majorities were married 347(95.3%); are from out of Jimma town 290 (77%), and had no formal education 190(52.2%). Eighty five percent of the cases have visited other institutions and arrived to JUTH with referral paper.

**Table 1:** Sociodemographic and obstetric characteristics of women with severe maternal morbidity and severe maternal outcome, South-west Ethiopia

Characteristics		Severe maternal morbidity (N, %)	Severe maternal outcome (N, %)	Total (N, %)
		202(55.5)	162(44.5)	364(100.0)
Age in years	15–24	67(33.2)	34(21.0)	101(27.8)
	25–34	111(54.9)	94(58.0)	205(56.3)
	≥35	24(11.9)	34(21.0)	58(15.9)
Residence	Jimma Town	56(27.7)	18(11.1)	74(20.3)
	Out of Jimma Town	146(72.3)	144(88.9)	290(79.7)
Current marital status	Married	192(95.0)	155(95.7)	347(95.3)
	Single/separated/divorced	10(5.0)	7(4.3)	17(4.7)
Educational status of the woman	No formal education	87(43.1)	103(63.6)	190(52.2)
	Primary School	63(31.2)	38(23.5)	101(27.7)
	High school & above	52(25.7)	21(12.9)	73(20.1)
Gravidity	I	84(41.6)	34(21)	118(32.4)
	II-IV	69(34.2)	60(37)	129(35.5)
	≥V	49(24.3)	68(42)	117(32.1)
ANC follow up	Health center	138(68.3)	114(70.4)	252(69.2)
	Hospitals	30(14.9)	13(8.0)	43(11.8)
	Private clinics	6(3.0)	4(2.5)	10(2.8)
	No ANC follow up	28(13.9)	31(19.1)	59(16.2)
Referral status	Yes, referred from other facility	162(80.2)	149(92.0)	311(85.4)
	No	40(19.8)	13(8.0)	53(14.6)
Date of admission	Holiday/weekends	42(20.8)	39(24.1)	81(22.3)
	Workdays	160(79.2)	123(75.9)	283(77.7)
Mode of delivery^c^ (*n* = 362)	All forms of vaginal delivery	82(22.7)	60(16.6)	142(39.2)
	Cesarean section	93(25.7)	42(11.6)	135(37.3)
	Laparotomy	6(1.7)^a^	46(12.7)^b^	52(14.4)
	All forms of abortion	21(5.8)	12(3.3)	33(9.1)

^a^All are cases of Ectopic Pregnancy; ^b^40-Uterine Rupture, 3-Ectopic Pregnancy and 3-Gestational trophoblastic disease cases; ^c^ Two cases died while pregnant

About 65% of the mothers were pregnant either for the first time or more than four times. Although, the large majority (84%) of the mothers had ANC follow up, only half of them had three or more visits. More than three-fourth of them gave birth to the index pregnancy at the study facility. Of the remaining: 38(10.4%) came with complication after giving birth at home and the remaining arrived with complications after giving birth at other institutions. Two mothers were still pregnant at the time of death. The commonest mode of delivery among all cases was vaginal (all forms of vaginal delivery) in 142(39%). On the other hand, C/S was the most common mode of delivery in woman with SMM and/or SMO who gave birth after arriving JUTH, accounting for 131(45.8%) of them. Laparotomy was performed in 52(14.4%) of the cases of which uterine rupture cases were 40 and the rest were cases of ectopic pregnancy and gestational trophoblastic diseases.

### Facility obstetric care indicators

Based on WHO MNM criteria 50% of the cases of SMO fulfilled more than one criterion for the organ dysfunction. The most common organ dysfunction was uterine in 42.6%, followed by respiratory in 29.6%, and cardiovascular in 27% of SMO cases. Twelve 7.4% of these mothers had renal dysfunction with five of them referred out to Addis Ababa for dialysis and the rest seven died before such arrangement was made.

As presented in Table 2, the majority of SMO cases; 71.6% had evidence of organ dysfunction on arrival to the JUTH or within 12 h arrival with 12 of these being MD. Interestingly 96.6% of these cases came referred from other health facilities. Forty-six of the cases developed SMO condition after 12 h of hospital stay making the intra-hospital SMO rate of 16.8 per 1000 live births. Critical interventions were performed in 52.2% of all cases with SMM or SMO. The interventions were: blood transfusion in 40.7%; laparotomy in 22.5%; and ICU admission 6.6% of all cases of SMM and SMM (Table 2).

**Table 2:** Indicators of severe maternal morbidity, severe maternal outcomes and facility care in South-west Ethiopia

Characteristics	Number of cases	Proportion
Cases with severe maternal morbidity and severe maternal outcome	364	13.3%
Women with severe maternal morbidity (per 1000 live births)	202	73.8
Severe maternal outcome ratio (per 1000 live births)	162	59.2
Maternal mortality ratio (per 100,000 live births)	24	876.9
Maternal near-miss ratio (per 1000 live births)	138	50.4
Hospital access indicators		
Severe maternal outcome identified on arrival or within 12 h of hospital stay	116	71.6%
Mothers who died on arrival or within 12 h of hospital stay	12	50%
Severe maternal outcome cases referred to the study facility	112	96.6%
Intra-hospital care		
Transfusion per total live births	148	5.2%
Intensive care unit admission per total live births	24	0.9%
Intensive care unit admission among women with severe maternal outcome	24	14.8%
Cases of maternal death managed in the ordinary ward (out of intensive care unit)	14	58.3%
Laparotomy per total live births	82	2.9%
Rate of intra-hospital severe maternal outcome (per 1000 live births)	46	16.8
Patients referred out from Jimma University Teaching Hospital (per 100 cases of severe maternal outcome)	5	3.0%
Intra-Hospital deaths	12	50%

### Complications underlying severe maternal morbidity and severe maternal outcome

Table 3 shows the distribution of complications underlying SMM and SMO. The three leading obstetric complications identified were: hypertensive disorders in 32.7%, obstetric hemorrhage in 27.5%, and pregnancy-related infections in 13.5%. On the other hand, among cases of MNM cases, uterine rupture in 29.7%, obstetric hemorrhage in 22.5% and severe hypertensive disorders in 21% were the most common underlying complications. Early pregnancy complications accounted for 13.4 and 10.9% of cases of SMM and MNM but no case of maternal death were attributed to these complications. Except for three cases of indirect maternal deaths; all of the maternal deaths were direct obstetric deaths. Eclampsia, postpartum hemorrhage and pregnancy-related infections were the top three leading causes of MD in this study: each accounting for 29.2, 20.8 and 12.5% of the 24 maternal deaths. The highest MI was associated with eclampsia followed by antepartum and then postpartum hemorrhage.

**Table 3:** Morbidity conditions among women with severe maternal morbidity and severe maternal outcome in South-west Ethiopia

Complications	Total eligible			Mortality Index
	Severe Maternal Morbidity	Severe Maternal Outcome		
		Maternal near-miss	Maternal deaths	
	*N* = 202(%)	*N* = 138(%)	*N* = 24(%)	14.8
Obstetric hemorrhage	62(30.7)	31(22.5)	7(29.1)	18.4
ـ Postpartum hemorrhage	27(13.4)	23(16.7)	5(20.8)	17.9
ـ Antepartum hemorrhage	35(17.3)	8(5.8)	2(8.3)	20
Hypertensive disorders	81(40.1)	29(21)	9(37.5)	23.7
ـ Severe preeclampsia	71(35.1)	11(8)	2(8.3)	15.4
ـ Eclampsia	10(5)	18(13)	7(29.2)	28
Pregnancy related infections	32(15.8)	14(10.1)	3(12.5)	17.6
Uterine rupture	–	41(29.7)	2(8.3)	4.7
Early pregnancy complications	27(13.4)^a^	15(10.9)^b^	–	–
Others	–	8(5.8)	3(12.5)^c^	27.3

^a^Septic abortion 12(5.9%); Abortion related hemorrhage 9(4.5%); & Ectopic pregnancy 6(3%); ^b^Septic abortion 3(2.2%), Abortion related hemorrhage 3(2.2%), Ectopic pregnancy 3(2.2%) & Molar pregnancy 6(4.3%); ^c^Poisoning during pregnancy, Hepatitis and Advanced AIDS each

### Factors contributing for severe maternal morbidly and severe maternal outcome

**Fig. 1:**
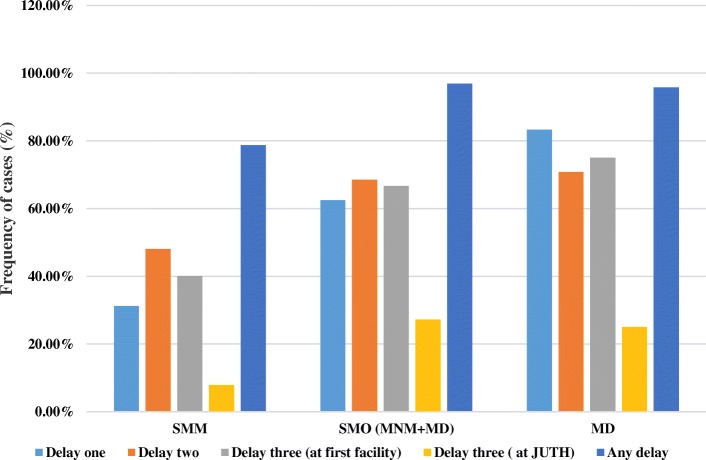
Frequency of occurrence delay to care in relation to severity of maternal complications; Southwest Ethiopia (SMM: severe maternal morbidities, SMO: Severe maternal outcome, MNM: maternal near-miss, MD: maternal death, JUTH: Jimma University Teaching Hospital

Preventability of SMM and the SMO was assessed by identifying factors contributing to the delay in receiving appropriate care using the three delay model (Table 4). There were delays at the three levels inhibiting the cases from receiving an appropriate care and thus contributing to the severity of complications. Moreover, the frequency of cases identified to have a delay by the level of occurrence of the delay was found to be more and more frequent in line with the degree of severity of maternal complications; as we go from SMM to MNM and then MD (Fig. 1). Overall 86.8% of SMM and SMO cases have encountered some form of delay in the continuum of care. Delay in seeking care (delay one), reaching at the appropriate health facility (delay two) and receiving appropriate care after reaching at the health facility (delay three) were identified among 45.1, 57.1 and 59.1% of SMM and SMO cases. More than half 51.9% of all cases had encountered delay three at the referring or first health facility visited (Table 4).

**Table 4:** Reasons for delay among women with severe maternal morbidity and severe outcome in South-west Ethiopia

Reasons for the delay in receiving an appropriate care	N (%)
Patient/family related factors	
ـ Absence of ANC^a^ after second half of pregnancy	46(12.6)
ـ Symptom/labor at home for more than twelve hours	97(26.6)
ـ Arriving with complication after home delivery	38(10.4)
Total number of women with delayed seeking care (delay one)	164(45.1)
Delay two (Delay in reaching at the right facility)	
ـ Distance (> 40 km) from final facility	175(45.1)
ـ Transportation problem	86(23.6)
Total number of women with delayed access to appropriate facility (delay two)	208(57.1)
Delay three (delay after reaching a health facility)	
At the initial facility visited (among referral cases)	
ـ Referred without initiation of treatment needed	173(55.3)
ـ Other referral problems	105(33.8)
ـ Total number of women with delay at first facility	189(51.9)
Delay three at the study hospital	
ـ Professional (follow up, evaluation, communication)	19(5.2)
ـ Logistics (ICU^b^-bed, blood, service, drugs, lab. reagent)	41(11.3)
ـ Total number of women with delay at JUTH (delay three)	60(16.5)
Total number of women with delay at health facility (delay three)	215(59.1)
Total number of women with any delay	316(86.8)
Means of transportation on presentation to the study hospital	
ـ Public ambulance	237(65.1)
ـ Paid transportation	110(30.2)
ـ Other	17(4.7)

^a^*ANC* Antenatal Care, ^b^ *ICU* Intensive care Unit

### Reasons for the delay

The distributions of reasons for the delay for appropriate care by the level delay are shown in Table 4. Delay in seeking care after the onset of danger signs and symptoms of pregnancy including onset of labor was the most frequent patient related factor whereas, delay in reaching at the appropriate facility (delay two) was found to be mainly because of the long distance to the health facility. The commonest means of transportation used in arriving to JUTH was public ambulance being used by 65.1% of the cases. Close to one-fourth of the cases encountered difficulty of accessing means of transportation timely and they were obliged to use public transportation, contracted vehicle or waited for long in the referring facility for the public ambulance.

Fifty five percent of women who arrived with referral from other institution had no any treatment initiated on transfer to JUTH. Other referral problems were identified in 33.8% of all cases. These included keeping women with high risk conditions like previous uterine scar in a setting without cesarean facility and referral of critical cases without accompaniment by health professionals. In addition delay in getting appropriate care after reaching at JUTH was identified in one out of six cases (Table 4).

### Factors associated with severe maternal outcome

Statistically significant association was observed between SMO and maternal age, residential area, educational level of the woman and occupation (*P* < 0.0001). Among obstetrical variables: gravidity, number of ANC visits, referral status, the state of pregnancy at the time of complication, mode of delivery and the duration of hospital stay were found to be associated with SMO (*p* < 0.05). The marital status and place of ANC had no significant associated with SMO on univariate logistic regression.

When binary logistic regression analysis for the determinants of SMO carried out none of the background variables remained significant after adjusting for all the variables in the list. On the other hand, complications identified for the first time during intrapartum period (AOR = 2.66(1.29, 5.47)) and prolonged hospitalization (> 7 days) (AOR = 2.77(1.42, 5.42)) increased the odds of the SMO. Gravidity and referral status did not show statistical significance whereas the optimal number of ANC visits is shown to be protective of SMO: it reduces such outcome by nearly half (AOR = 0.52(0.29, 0.91)) compared to women with no or suboptimal ANC visits. Interestingly, compared to all other modes of delivery, women delivered by caesarean section are also less likely to develop SMO, (AOR = 0.43(0.24, 0.79)). In addition, all forms of delays increased the odds of severe maternal outcomes with delay one (AOR = 2.37(1.36, 4.12)), delay two (AOR = 2.66(1.39, 5.07)) and delay three (AOR = 4.12(2.34, 7.26)) after adjusting for all variables in the list (Table 5).

**Table 5:** Binary multivariable logistic regression analysis of determinants for severe maternal outcome in South-west Ethiopia

Characteristics		SMM^d^ (%)	SMO^n^ (%)	Crude odds ratio (95% CI)	Adjusted odds ratio (95% CI)
Age group in years	< 25	67(33.2)	34(21)	1	1
	25–34	111(55)	94(58)	1.67(1.02,2.74)*	1.22(0.65,2.29)
	≥35	24(11.9)	34(21)	2.79(1.43,5.43)*	2.04(0.78,5.34)
Residence	Jimma town	56(27.7)	18(11.1)	1	1
	Out of Jimma town	146(72.3)	144(88.9)	3.07(1.72,4.74)*	0.92(0.32,2.63)
Education status	No formal education	87(43.1)	103(63.6)	2.93(1.64,5.22)*	0.75(0.26,2.17)
	Primary School	63(31.2)	38(23.5)	1.49(0.78,2.85)	0.72(0.29,1.75)
	High school & above	52(25.7)	21(12.9)	1	1
Occupation	Farmer	101(50)	118(72.8)	1	1
	Public employee	36(17.8)	18(11.1)	0.43(0.23,0.8)*	1.81(0.58,5.64)
	Merchant	51(25.2)	15(9.3)	0.25(0.13,0.48)*	0.48(0.19,1.18)
	Others^a^	14(6.9)	11(6.8)	0.67(0.29,1.55)	0.71(0.23,2.14)
Gravidity	Primigravida	84(41.6)	34(21)	1	1
	Gravida II-IV	69(34.2)	60(37)	2.26(1.44,3.54)*	2.08(1,3.77)
	Gravida ≥V	49(24.3)	68(42)	3.43(1.99,5.89)*	1.9(0.8,7.42)
State of pregnancy at detection complication	Antepartum	56(27.7)	24(14.8)	1	1
	Intrapartum	68(33.7)	75(46.3)	2.57(1.44,4.59)*	2.66(1.29,5.47)**
	Postpartum	51(25.2)	47(29)	2.15(1.16,4.02)*	1.79(0.83,3.86)
	Post-abortion	27(13.4)	16(9.9)	1.38(0.63,3.02)	1.04(0.38,2.58)
Optimal ANC visits		110(54.5)	50(30.1)	0.37(0.24,0.58)*	0.52(0.29,0.91)*
Referred from other facility		162(80.2)	149(92)	2.83(1.48,5.5)*	1.06 (0.42,2.69)
Delivery by emergency Cesarean Section		93(46)	42(25.9)	0.41(0.26,0.64)*	0.43(0.24,0.79)**
Hospital stay (> 7 days)		175(86.6)	110(67.9)	3.06(1.82,5.17)*	2.77(1.42, 5.42)**
Delay one		63(38.4)	101(61.6)	3.65(2.36,5.65)*	2.37(1.36,4.12)**
Delay two		97(46.6)	111(53.4)	2.36(1.53,3.63)*	2.66(1.39,5.07)**
Delay three		128(77.2)	37(22.8)	4.20(2.65,6.66)**	4.12(2.34,7.26)**

*and ** significant at 5 and 1% level of significance respectively; ^a^ others include housewife, unemployed and students. ^d^severe maternal morbidity; ^n^severe maternal outcome

## Discussion

This prospective cross-sectional study of woman with various pregnancy related complications using the near-miss approach to maternal health is first of its kind in the study institution. Our study has found that the burden of maternal ill-health in population the hospital serves is high. In addition the finding of high proportion of complication already present on arrival to JUTH indicates the significance of delay at the different level of care. Our study reaffirmed this theory. Unlike many other studies on near-miss, our study has compared women with extremely severe outcome to woman with less severe outcome and identified significant contributors to their severity. However, comparison of our study findings with that of other similar institutions in the country may not be enough because of the paucity of studies done using this approach of maternal health assessment.

Our study showed the ration of SMO incidence and MNM incidence to be 59.2 and 50.4 per 1000 live births. The MMR was 876.9 per 100,000 live births whereas, the MI was 14.8%. In general, the rate of occurrence of SMO indicators were found to is higher than the findings of earlier study done in other part of the country [9, 14, 16] and other developing countries [10, 11, 13, 17–24]. Moreover, the MMR was higher than the national average MMR report for the year 2013 [1]; and the findings of earlier facility based study done elsewhere in the country [14, 15, 25–29], and that of other developing countries [11, 13, 19–22, 30]. This high incidence ratio might be due to the fact that our study facility is the only referral hospital in south-west region of the country serving complicated cases referred from other health facilities in its catchment. Moreover, the occurrence of a high rate of intra-hospital SMO and MI, higher than the findings from otter facilities in the country [31] and other developing countries [19] puts the quality of obstetric care offered to women with obstetrical complications in JUTH under question.

Our study showed that direct obstetric causes were the most common underlying factors of SMM and SMO. The most common underlying morbidity of SMM were hypertensive disorders and obstetric hemorrhage similar to the findings of the study done elsewhere in the country [16] and other developing countries [19, 22]. However, uterine rupture, obstetric hemorrhage and hypertensive disorders were found to be the top leading underlying complications among cases of SMO and this is comparable to the findings from studies in other parts of the country [9, 14, 25, 31] and other developing countries [10, 13, 17–24] and sub-Saharan countries [32].

Of the total of 24 MD, 21 of them died of direct obstetric causes and the rest three were indirect maternal deaths comparable with earlier studies elsewhere in the country [14, 15, 26] other developing countries [11, 13, 17, 22, 24, 33]. Our study showed the leading causes of MD to be hypertensive disorders followed by obstetric hemorrhage and this finding is in agreement with the findings of earlier study done in different hospitals from Ethiopia as a part of FIGO logic initiative and the review of maternal mortality trend in Ethiopia [14, 26]. Eclampsia was associated with the highest case fatality; similar to the findings of earlier studies done in other parts of the country [9, 14] and other developing countries [17, 21, 32].

Unlike earlier studies in the same facility and other parts of the country [15, 26, 28], uterine rupture is no more the leading cause of MD. This could be due to improved care for uterine rupture cases at the facility. The fact that 30 out of 43 (70%) cases of uterine rupture cases presented after laboring at home for over 12 h show the delay in seeking care. Uterine rupture as the leading causes of MNM. This is in contrast to the findings from earlier studies in this and other health facilities in the country [15, 26, 29, 34]; in our study there was no case fatality because of complication from abortion supplementing the findings of study stating case fatality from complications of abortion in Ethiopia was declining [27, 28]. Perhaps this could be attributed in part to the recent ‘revision of abortion law and its legislation’ in the country which ensures the provision of safe abortion services for selected group of women, avoiding fatal complications from unsafe abortion [35].

Emergency obstetric care use by women is influenced by a complex interaction of factors leading to delay in decision-making, accessing services and receipt of proper care once a health facility is reached [1, 5, 18, 24, 30, 36]. Similar to the case of our study; delay one, delay two and delay three were reported as a significant contributor to SMO in several earlier studies from developing countries [10, 18, 30, 36]. In this study, the occurrence of delay one, two and three was 45.1, 57.1, and 59.1% respectively, similar to studies done elsewhere in the country [9, 14, 29]. Delay-1 (delay in seeking health care) is seen more frequently than the findings from other developing countries [37] however it is better than the finding of earlier studies done in Ethiopia [9, 14]. This can be justified by a difference in socio-demographic characteristics of the study population and the year of study.

According to WHO the fact that large number women arrived to health care facility with SMO indicate occurrence of the first (delay in recognizing a condition as a complication and delay in seeking help) and second (delay in reaching a health-care facility once the decision to seek care has been made) delays in the health district [5]. This fact is well supported by the findings of our study. In our study, close to three-fourth of women had SMO on admission or within twelve hours of arrival. This finding is also in line with studies from other institutions in Ethiopia and other developing countries [11, 14, 16, 28, 31].

In this study, delay three (at first and/or last facility) was found to have the strongest association with SMO: with a four-fold increase in the risk. This supports the WHO hypothesis relating a high case fatality in the hospital as an indicator for the presence of delay in receiving an adequate and appropriate treatment [14, 37]. Seeking care from a facility that is ill-equipped to give emergency obstetric care contributes to significant delay even after reaching the health facility. These factors were reported as significant contributors of delay in several studies [25, 37, 38]. These non-functional health facilities are physically accessible and described as “physical obstacles” for pregnant women in accessing a functioning health facility in time [36]. In line with this, studies from northern Ethiopia attributed 88% of all maternal deaths to health system failure [29]. In our study, 77% of SMO cases, and 75% of the 24 MDs had health system related factors as a possible reason for delay three.

Almost all SMO on admission cases were transferred from other facilities, indicating presence of significant deficiency in the referral system. Large proportions of woman are kept unnecessarily before decision to refer is made. Even after deciding to refer these women, most are transferred without initiation of treatment. These can be due to professional factors (negligence or lack of skill) and/or possibly lack of the necessary supplies to provide the initial treatment, which are both not assessed in this study.

The fact that large number of woman reach to a facility with a complication indicates the complexity of care required by the population served by the health-care facilities in the assessment [5]. The complexity of care and treatment provided for obstetric patient ranges from basic to intensive care and thus the level of health facilities is different too [39]. The provision of appropriate health care for critically ill obstetric cases requires proper staffing, equipment, and management strategies and this health services obviously contribute to a better outcome among women with life-threatening conditions [24]. But, health facilities in the developing world are chronically under-resourced [24, 29]. Though most women presenting with organ dysfunction needs to be managed in ICU, the overall ICU admission rate of cases with SMO was low (14.8%) in this study. Moreover, only 40% of MD cases were managed in ICU because of lack of bed and/or mechanical ventilators. Additionally, lack of dialysis was the other reason for the delay at the study facility. Cases requiring dialysis were referred out to the hospital located in the capital of the country many kilometers away. Of the total of 12 cases with renal dysfunction and therefore requiring dialysis five were referred out and seven died before such arrangement is made. This supports the WHO hypothesis relating a high case fatality in the hospital as an indicator for the presence of delay in receiving an adequate and proper treatment [5, 24].

In general, logistic related problems like: lack of blood products, scarcity of ICU beds, unavailability of dialysis, laboratory reagents, and drugs are responsible for most of SMO with delay three (in JUTH) in our study. This is in line with the finding of the recent systemic review of delay-three, which highlighted its importance in developing nations. In that review, professional (inadequate training/skills mix (86%); staff shortages (60%); low staff motivation (44%) and logistic problems like drug procurement/ logistics problems (65%); lack of equipment (51%)) were identified as the most common barriers [24, 37]. In contrast, professional factors were less prominent in the case of delay three in JUTH. This can be explained by the fact that; the study is conducted in a teaching hospital with different level professionals and strict morning meeting discussion and feedback system.

The tendency of seeking reproductive health services, including family planning and ANC by the woman is affected by different socio-demographic factors and cultural barriers. Uneducated women are less likely to use the services. They may underestimate the value of institutional delivery or their decision power is limited in the male dominant community. As such their health seeking behavior will be limited (delay one) [24, 40, 41]. The fact that only 10% of deliveries in Ethiopia occurred in a health facility is clear evidence for this. In addition, more than 50% of women have no any formal education reported in Ethiopia [38] similar to the finding from our study and with other studies done elsewhere in the country [14, 31], but lower than that of other developing countries [10].

In our study, the tendency of booking for ANC seems to be high, but the proportion of cases with an optimal number of ANC visits was low and it is lower than the finding from other developing countries [10, 18, 23]. This difference could be because of the difference in education status. Similar to studies in other part of the country [9, 14, 31] and other developing countries [10, 18] residential area, educational status and occupation, optimal number of ANC visits, state of pregnancy at the time of the first detection of complication, mode of delivery index pregnancy and duration of hospitalization were found to associated with SMO in our study. Findings, which have significant implications for interventions in our study include: an adequate number of ANC and delivery by C/S being protective of SMO, similar to the findings from earlier studies [40].

Even if C/S is believed to increase the risk of developing SMM or MNM because of the associated increased risk of infection and hemorrhage, the rate of C/S is expected to be higher among cases of SMO for the obvious reasons [10, 24, 42]. The rate C/S among cases of SMO was high in our study similar to the study in other hospitals elsewhere in the country [9], and other developing countries [13]. In our study, C/S was found to be protective of SMO as the case study done in China [33]. This is, however, different from findings in studies from other developing countries which have found increased risk of SMO in women who underwent C/S [10, 24]. The higher incidence of uterine rupture (24.7%) in this study is higher than the report in those studies and can possibly account for the difference. In adition, the populations studied, the setting and delays identified at different level can also explain this difference.

This study, undertaken near the end of MDG and eve of SDG, showed an alarm to the situation of maternal health care in the study facility and hence the referral facilities and the population it serves. The high prevalence of delay in receiving an appropriate care after reaching the facility needs an urgent solution. We have witnessed cases of pregnant mothers being admitted and followed for labour in the referral facility whereas, these cases were beyond the capacity of those facilities to manage such cases like: the case of transverse lie being admitted and followed in labour for more than 24 h and later referred with uterine rupture, a mother with two C/S scars referred with uterine rupture after 2 months stay at maternity waiting homes of those health facilities and a case of cord prolapse where the cord was clamped, cut and legated before transfer, among others. The fact that more than half the cases with severe complication lacking formal education and the delay in seeking care needs to be addressed by the government and the health system. We feel that the finding of this study may pass a strong message to all parties who work to end preventable maternal deaths at the study area. The preventive effect of the ANC and C/S, mentioned above, for SMO can only be used by the majority only if this system is in place and close to the community. In addition, the significant contribution of delay three to MNM and maternal death calls for action.

## Conclusion

In conclusions, the burden of severe maternal outcome is high at JUTH, southwest Ethiopia. Preventable and/or treatable direct obstetric causes are responsible for the development of these maternal complications among the majorities of mothers with severe complications. Our study highlights the persistence of delays at all levels and especially delay three and its contribution for the severity of maternal complications. Moreover, this study has also indicated the protective effect of having the minimum recommended number of ANC visit and emergency C/S from the development of SMO.

We recommend that strategies for reduction of MNM and MD might be best achieved by implementing strategies for the prevention or reduction of delays in proper care provision to obstetric cases. It is our strong believe that, apart from enhancing obstetric cares like ANC and comprehensive emergency care, provision of training to maternal health service providers at the referring facilities needs to be considered. In addition the health care provided to mothers with SMM and critically ill obstetric patients at the tertiary hospital needs to be assessed and the logistic problems identified in the study institution should be timely addressed.

### Strengths and limitation:

Our present study used a prospective data to assess the situation of SMO at the university teaching hospital and the care provided to women with potentially life threatening and SMO conditions using a validated WHO tool for MNM.

This study may also have limitations for it has been conducted in a single tertiary hospital and the results might not be representative of other institutions and the community. However, to obtain additional evidence to refute or support the existence of an association between suspected cause and maternal outcome after discharge prospective cohort design have more power but with high cost and time.

## Abbreviations

ANC: Antenatal care
C/S: Cesarean section
CI: Confidence interval
FIGO: International federation of gynecology and obstetrics
ICU: Intensive care unit
JUTH: Jimma University teaching hospital
LTCs: Life-threatening complications
MDGs: Millennium development goals
MDSR: Maternal death surveillance and response
MI: Mortality index
MMR: Maternal mortality ratio
MNM: Maternal near miss
PLTCs: Potentially life-threatening conditions
SDG: Sustainable development goal
SMM: Severe maternal morbidity
SMO: Severe maternal outcome
WHO: World health organization
